# Altered Metabolism in Motor Neuron Diseases: Mechanism and Potential Therapeutic Target

**DOI:** 10.3390/cells12111536

**Published:** 2023-06-02

**Authors:** Cassandra Barone, Xin Qi

**Affiliations:** Department of Physiology and Biophysics, School of Medicine Case Western Reserve University, Cleveland, OH 44106-4970, USA; cmb308@case.edu

**Keywords:** Motor Neuron Diseases, metabolism, mitochondria, skeletal muscle

## Abstract

Motor Neuron Diseases (MND) are neurological disorders characterized by a loss of varying motor neurons resulting in decreased physical capabilities. Current research is focused on hindering disease progression by determining causes of motor neuron death. Metabolic malfunction has been proposed as a promising topic when targeting motor neuron loss. Alterations in metabolism have also been noted at the neuromuscular junction (NMJ) and skeletal muscle tissue, emphasizing the importance of a cohesive system. Finding metabolism changes consistent throughout both neurons and skeletal muscle tissue could pose as a target for therapeutic intervention. This review will focus on metabolic deficits reported in MNDs and propose potential therapeutic targets for future intervention.

## 1. Introduction

Motor Neuron Diseases (MND) are characterized by the progressive loss of motor neuron function due to untimely death of upper (UMN) and/or lower (LMN) motor neurons [1]. The mechanism driving the motor neuron degeneration is currently unknown; however, studies examined further in this review reveal that metabolic malfunction could be a leading cause. These alterations in metabolism expand beyond the central nervous system (CNS) and have been noted in the neuromuscular junction (NMJ) as well as skeletal muscle in patients with Amyotrophic Lateral Sclerosis (ALS) [2]. Declining NMJ integrity points to a neuron-to-muscle communication error in motor neuron diseases, proposing a greater need to study the interaction between these two systems [3,4,5,6]. Specifically, determining the changes of varying metabolic pathways such as glycolysis, the pentose phosphate pathway (PPP) and oxidative phosphorylation, both in and out of the CNS, is a top priority in the field. Therefore, understanding metabolism and recognizing alterations in MNDs is important in uncovering disease pathogenesis.

## 2. Types of MNDs

There are numerous different motor neuron diseases currently classified throughout the world. However, some of the most commonly known are ALS, Primary Lateral Sclerosis (PLS), Spinal Muscular Atrophy (SMA) Kennedy’s Disease, and Hereditary Spastic Paraplegia (HSP). Differences between these diseases rely on the types of motor neurons affected. Specifically, ALS is defined by loss of both upper and lower motor neurons [7], whereas PLS affects primarily upper motor neurons and SMA targets lower motor neurons [8,9]. It is important to understand the differences between these diseases in order to be able to properly diagnose and treat patients once the disease phenotype becomes apparent.

### 2.1. Amyotrophic Lateral Sclerosis

ALS was originally characterized in 1869 by Jean-Martin Charcot; however, it wasn’t until the diagnosis of the famous New York Yankee in 1939, Lou Gehrig, that the disease become more well-known [10]. Generally, the disease is characterized by the loss of both upper and lower motor neurons. Clinically, disease onset presents as either bulbar onset, in which patients suffer from more proximal weakness presented as dysarthria and dysphagia, or limb onset, in which patients suffer from distal weakness in limbs [11,12]. Although presentation of disease onset varies, most patients ultimately succumb to respiratory failure resulting in mortality [13,14].

The prevalence of ALS is estimated to be around 6 cases per 100,000 people globally [15]. The mean age of onset is between 50–66 years of age, with variation between continents [15]. Although there is a wide range of diagnosis age, typical time to mortality is between 3–5 years for all cases due to time to diagnosis after initial phenotype is shown [16]. Among diagnosed ALS patients, about 90% of cases have an unknown origin and are defined as sporadic ALS, whereas 10% are genetically inherited variations denoted as familial ALS [17]. Although the mechanism of sporadic cases has yet to be discovered, numerous genes associated with disease pathology have been associated with the familial form of ALS [18]. In 1993, the first gene, super oxide dismutase (SOD1), was associated with ALS [19]. The primary function of SOD1 is to reduce oxidative stress by reducing free radicals; however, in ALS, both wild-type (WT) and mutant (MT) SOD1 are prone to misfolding and ultimately aggregation [20]. Since the discovery of SOD1 and its relation to ALS, genes such as transactive response-DNA binding protein (TARDBP) [21], fused in sarcoma (FUS) [22] and C9orF72 [23] have been of primary focus in ALS research.

### 2.2. Primary Lateral Sclerosis

Since its discovery, PLS has been denoted a primarily upper motor neuron disease. In 1874, Jean-Martin Charcot’s original examination confirmed the unreliable nature of affected lower motor neurons [24]. In the early 20th century, studies began to show a predominant loss of upper motor neurons along with decreased degeneration present in the anterior horn, which would classify the disease under ALS [25,26]. Since then, diagnostic criteria and methods have been refined by utilizing both magnetic resonance imaging (MRI) for degeneration analysis and positron emission tomography (PET) for total glucose uptake [27]. The question remains as to whether PLS will eventually evolve into ALS over time. In order to determine disease course, 43 patients with PLS were analyzed over the course of nine years. [28]. The results showed those with pure PLS at the time of diagnosis did not go on to develop ALS, emphasizing differences in the diseases and the need to determine mechanisms regarding disease phenotype. Although mean age of onset is similar to ALS, PLS only represents about 3% of motor neuron disease patients, making it a rare but sporadic disease [29].

As previously stated, PLS has been denoted as a primarily sporadic disease to differentiate it from hereditary spastic paraplegia (HSP) and aid in diagnostic criteria [30]. However, patients with a genetic history of familial ALS have exhibited PLS phenotypes, suggesting, although rare, a potential genetic cause of this previously primarily sporadic disease [31]. The rare nature of PLS continues to be emphasized in the small number of juvenile cases that have been reported. Juvenile PLS is a genetic variation that occurs before the second decade of life. Mutations in the ALS2 gene encoding the protein alsin [32] and the ERLIN2 gene encoding the protein endoplasmic reticulum lipid raft-associated protein 2 [33] have been recorded in juvenile PLS cases, creating more layers to an already complicated disease.

### 2.3. Spinal Muscular Atrophy

SMA is an autosomal recessive disease targeting both motor neurons and skeletal muscles [34]. The current rate of incidence reported for SMA is about 1 in every 10,000 births [35]. In 1995, the gene survival motor neuron (SMN) was discovered to be the primary cause of SMA [36]. Two variations of this gene are found in the human genome, SMN1 and SMN2, where SMN1 is present in all mammals and SMN2 is specific to humans [37]. Mutations in SMN1 have been found in over 90% of SMA cases, making it the primary target for therapeutic intervention [38].

SMA can be characterized into five types, varying by both age and severity. Typically, the disease presents as proximal weakness of the legs and minimal effects on upper extremities [39]. Although this disease has presented itself later in life, most cases begin to show signs as early as 18 months of age [40]. Those presenting with prenatal signs typically succumb to the disease within 1 month of birth [41]. However, cases diagnosed later in life, though rare, show minimal disease phenotype as well as showing no alterations in life span [40]. Therefore, the field is currently in need of a way to tackle this disease at early stages.

### 2.4. Kennedy’s Disease

Otherwise known as spinal and bulbar muscular atrophy (SBMA), Kennedy’s disease is an X-linked recessive disease characterized by CAG repeats in exon 1 of the androgen receptor gene [42]. This MND is different from others as it only presents in male patients. The disease typically presents in the lower limbs; however, simultaneous limb motor deficits have been shown in this disease [43]. Although progressive atrophy is a hallmark of this disease, life expectancy remains unchanged in comparison to the normal population in those diagnosed with the disease [44]. Therefore, finding early stage biomarkers is necessary for proper therapeutic intervention.

### 2.5. Hereditary Spastic Paraplegia

HSP is a group of inherited heterogeneous diseases clinically defined by both stiffness and weakness in the lower limbs [45]. HSP can be classified into numerous subtypes based on symptoms, age, inheritance pattern or cellular mechanism [46]. Clinical classification is defined by either pure or complicated signs [46]. Pure HSP presents with spastic paraplegia and lower extremity hypoesthia [47]. Complicated HSP consists of neurological and non-neurological symptoms such as dysarthria and peripheral neuropathy, in addition to all features present with pure HSP [47]. Age of onset for the disease ranges from early infancy to late adulthood, with symptoms before the age of 35 denoting Type 1, and after, Type 2 [48]. Inheritance patterns recognize five specific types of HSP; autosomal dominant, autosomal recessive, X-linked, mitochondrial and de novo, which have been summarized previously [49].The corticospinal disease has an average prevalence of 4.5 in every 100,000 cases in the world [49]. Over 80 genes have been associated with HSP, 83 identified as spastic paraplegia genes, 25 having only HSP designation and 12 having yet to be associated with a specific protein [50]. With varying patterns of inheritance involving numerous pathways, there currently is a need to find common ground in order to effectively treat all forms of HSP [50].

## 3. Metabolism in Neuron-Muscle Communication

**Figure 1:**
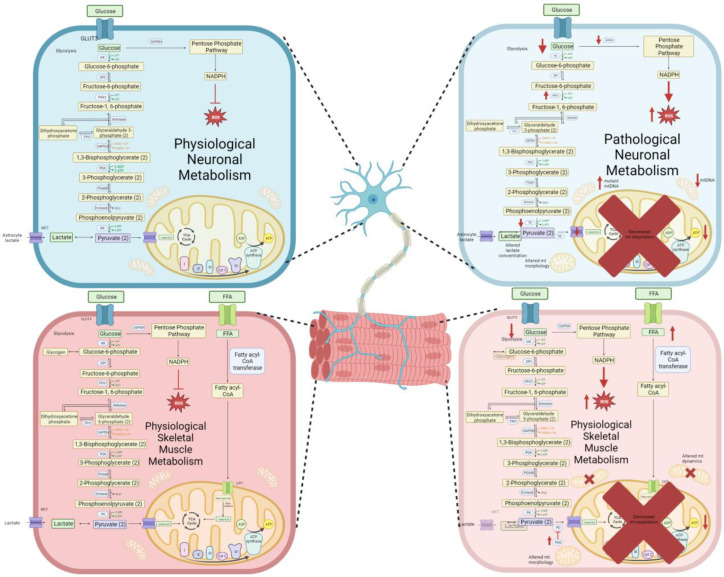
A summary of metabolism in neurons and skeletal muscle reflective of both physiological and pathological states. The panels on the left show normal metabolic output found in both cells types. Panels on the right emphasize the changes in metabolism within NMDs.

While numerous pathways have been proposed, metabolic dysfunction is heavily represented in MNDs. Studies currently reveal that altered metabolism could either be the cause or the result of disease pathogenesis [51]. Although motor neurons are a primary focus when investigating MNDs, similar metabolic dysfunction has been discovered in skeletal muscle tissue, including localization at the NMJ [52]. Therefore, understanding dysregulated metabolism in both the CNS and periphery is necessary for diagnostic and therapeutic intervention (Figure 1).

### 3.1. Neuronal

The CNS houses highly metabolic cell types requiring copious amounts of energy to maintain physiological functions. The intricate communication between cell types in order to maintain metabolic balance has been extensively reviewed [53,54]. The brain consumes about 20% of the body’s total energy in order to maintain proper functioning [54]. Glucose is the primary substrate utilized by neurons in the brain [55]. However, in states of low glucose availability, other substrates, such as ketone bodies, can be used as a substitute [56]. Under normal physiologic conditions, glucose is transported primarily into neurons via glucose transporters [57]. Glucose can then be phosphorylated, where its metabolic fate can be determined by either proceeding through glycolysis or being shunted into the pentose phosphate pathway (PPP) [58,59]. The switch between glycolytic pathway activation and PPP is determined by the metabolic needs of the cell. Shunting phosphorylated glucose into the PPP is necessary when needing to battle oxidative stress [60]. Conversely, glucose-6-phosphate continuing through glycolysis is needed in order to produce energy. The product of glycolysis, pyruvate, is either transformed into lactate within the cytoplasm or shunted to the mitochondria in order to be converted to acetyl CoA. Acetyl CoA is the primary substrate for the tricarboxylic acid cycle (TCA) [61]. The byproducts of the TCA cycle, NADH and FADH, are necessary for the mitochondrial electron transport chain (ETC) in order to produce the vast amount of ATP necessary to maintain cellular functions [61]. The mitochondrial ETC utilizes the complexes housed on the inner membrane of the mitochondria to create a proton gradient in order to produce ATP [62]. Defects in any of the complexes have been shown to result in decreased energy production in neurodegenerative disease, as described further throughout this text.

### 3.2. Neuromuscular Junction

Connections formed at the NMJ are important for muscle function and contraction. In short, an action potential is delivered to the synapse allowing for the release of acetylcholine (Ach); Ach can then traverse across the NMJ and bind to the Ach receptor on the muscle fiber, enabling a contraction downstream [63,64]. Metabolic balance at the NMJ is necessary for maintaining communication between neuronal and skeletal muscle cells [65]. It is currently known that mitochondria ATP production and neurotransmitter release at the NMJ is necessary to maintain a stable synaptic connection [66]. In a neuronal co-culture model, motor neurons show increased distal mitochondrial accumulation, emphasizing the highly metabolic nature of the synaptic connection between motor neurons and muscle fibers [67]. Although it is known that metabolism is a key regulator of this communication, more studies must be performed in order to determine the importance of the NMJ, more specifically metabolism at the NMJ, in motor neuron diseases.

### 3.3. Skeletal Muscle

Muscle cells primarily utilize glucose as an energetic substrate [68]. Glucose is transported across the cellular membrane and either stored as glycogen or directly metabolized through glycolysis [69]. Pyruvate shares a similar fate to neuronal metabolism, either being converted to lactate or proceeding through oxidative phosphorylation. Second to glucose, skeletal muscles can also use fatty acids in order to maintain energy homeostasis, primarily as a result of a high stress induced environment, such as exercise [70]. Conversely, in cases of aging muscle related disorders, such as sarcopenia, a decrease in total mitochondria via mtDNA and altered mitochondrial complex activity are key factors in muscle atrophy [71]. Basic mitochondrial functions such as fusion and fission have been linked to increased muscle atrophy in both age and disease [72], emphasizing the need for metabolic homeostasis in order to maintain muscle function.

## 4. Metabolic Dysfunction in Motor Neuron Diseases

### 4.1. CNS Metabolic Malfunction in ALS

A hallmark of ALS is disturbed energy homeostasis, defined by either increased resting energy expenditure, defined as hypermetabolism, or a decrease in energy production [51]. Evidence of hypermetabolism has been found in both sporadic and familial ALS [73]. Utilizing changes in fat mass and fat free mass, ALS patients were denoted as hypermetabolic in comparison to age match controls [51]. A 30-month follow-up confirmed decreased overall function via the Revised ALS Functional Rating Scale (ALSFRS-R) and survival in hypermetabolic ALS patients [51]. Upon diagnosis, ALS patients present with significantly lower weight at diagnosis compared to control patients [74]. Upon further review, patients with significant weight loss also had worse neurological function, emphasizing a direct correlation between disease progression and hypermetabolism [74]. Although hypermetabolism is a hallmark of ALS patients, malnutrition is also a consequence of a progressing diseased phenotype [75]. Malnutrition is aggravated by functional decline and creates a cycle of low supply and high demand, contributing to a worsening phenotype [75]. Previous studies note varying uptake of glucose throughout the CNS of ALS patients [76,77]. This results in regional specificity of glucose uptake, creating a decrease in glucose uptake in the motor cortex [77]. Along with decreased glucose uptake, numerous studies have consistently noted disrupted mitochondria in ALS patient samples [53,78,79,80,81]. Increased mutated mitochondrial DNA (mtDNA), as well as a decrease in standard mtDNA, was reported in ALS patient spinal cord samples [78]. Mutated mtDNA was increased in the motor cortex as opposed to the temporal cortex; however, this change was not noted in control patients [82].) Utilizing electron microscopy (EM), ALS patient lumbar spinal cord samples demonstrated altered mitochondria morphology [83,84]. Coupled with malfunctioning mitochondria, oxidative stress is a key factor in disease pathogenesis as shown in ALS patients [85]. Although there is evidence for mitochondrial malfunctions in ALS patients, not much has been proven about upstream metabolic pathways, such as glycolysis. Previous reports show a decrease in glucose uptake in ALS patient motor cortex via positron electron microscopy [76]. However, an increase in glucose utilization in astrocytes and microglia propose a cell-specific response to glucose utilization.

In vivo models of ALS mimic metabolic dysregulation within the CNS, as previously reported in patients. Since SOD1 was the first gene found to be associated with ALS, a significant amount of what has been found in vivo has come from this mouse model. SOD1 mice harboring the G93A mutation have shown defects in glucose uptake in the corticospinal tract, similar to PET studies in patients, as well as a decrease in total ATP production [86]. More importantly, transgenic mice expressing either hSOD1 WT or G93A mutant show a decrease in mitochondrial respiration and total ATP production at symptomatic stages of the disease [87]. Importantly, ALS has been shown to have a high prevalence in men over women, along with disease onset at an earlier age [88]. Utilizing the SOD1 G93A mouse model, a report recapitulates the sex differences seen in patients by emphasizing a delay in both weight loss and survival age post symptom onset [89]. Other ALS animal models have shown decreases in mitochondrial respiration. Utilizing a TDP43 animal model, increased TDP43 WT and mutant A315T expression showed increased mitochondrial malfunction and altered mitochondrial morphology, confirmed with transmission electron microscopy (TEM) [90]. Utilizing dissected motor cortices from TDP43-A315T mouse models, a decrease in both ATP/ADP and NAD+/NADH ratios confirms defects in mitochondrial respiration and increased oxidative stress, respectively [91]. A group led by Dr. Daniela Zarnescu further expanded the field of metabolic dysregulation of ALS by determining changes of glycolysis, the TCA cycle and mitochondrial respiration utilizing a *Drosophila* model. In TDP43 *Drosophila* models, the authors found that by increasing the glucose transport on neurons (GLUT3) as well as increasing one of the rate-limiting enzymes in glycolysis, phosphofructokinase (PFK), locomotive deficits can be restored back to baseline [92]. While an increase in this enzyme was already found in their model and increasing its presence mitigated the ALS phenotype, the authors proposed glycolysis could be acting as a compensatory mechanism in ALS and increasing its output could continue to eliminate locomotive defects. They continued to expand their research downstream of glycolysis to determine if altering metabolism at any state could be beneficial. Utilizing the same *Drosophila* model, they determined that increasing the TCA cycle by either genetic modulation or through dietary mechanisms could also eliminate locomotive deficits in this model [93]. Although other in vivo models have been created to further study ALS, minimal studies have emphasized the importance of metabolic malfunction, which should be emphasized moving forward to determine genetic differences in metabolic malfunction in ALS.

In vitro models of ALS offer a short-term solution for studying motor neuron diseases, and have the availability to study both familial and sporadic forms of the diseases. Importantly, one can not only investigate mitochondrial dysfunction, but also determine changes in other pathways such as glycolysis and the PPP to determine if there are any changes upstream of mitochondrial deficits that could alter the total metabolic output in these diseases. A study utilizing motor neurons derived from induced Pluripotent Stem Cells (iPSCs) were generated as isogenic controls, sporadic ALS (sALS) or familial ALS (fALS) [94]. The authors found, utilizing a seahorse respiration assay, that not only was glycolysis elevated in these motor neurons but also mitochondrial respiration was reduced. To confirm that this was specific to motor neurons, they differentiated the iPSCs into cortical neurons exhibiting the same genotype; however, no significant respiratory changes were found, suggesting this is a motor neuron specific response [94]. Similarly, a study utilizing fibroblasts isolated from ALS patients consisting of the SOD1 mutant I113T confirm similar results previously found in the G93A mutant, that mitochondrial respiration is decreased while glycolytic activation is increased [95]. 

Interestingly, these changes were observed in fALS motor neurons harboring SOD1, TDP43, and C9orf72 mutations; however, a study utilizing patient iPSCs with mutations in the ALS-causing gene FUS found no changes in metabolic respiration in comparison to isogenic controls, proposing a gene-specific metabolic response [96]. A separate study, utilizing motor neurons derived from E17 rat cortex infected with SOD1 and TDP43 mutations, showed decreased glycolytic activation by a reduction in lactate but maintained levels of pyruvate and ATP [97]. The authors postulate their results are representative of the short-term induction of ALS (48 h), hypothesizing that this data is an early-stage response and that end-stage ALS could show mitochondrial deficits as described previously. Focusing on the PPP, a previous study notes a decrease of ribose-5-phosphate and glucose-6-phosphate dehydrogenase (G6PDH) resulting in an increase of oxidative stress in an SOD1 animal model of ALS [98]. This emphasizes alterations in numerous neuronal metabolic pathways in ALS models (Figure 1 and Table 1).

### 4.2. Peripheral Metabolic Malfunction in ALS

In recent years, skeletal muscle metabolism has been evaluated in motor neuron diseases in order to properly determine a therapeutic course of action for disease treatment. ALS patient skeletal muscle tissue samples reflect what was previously shown in the spinal cord samples. In a study utilizing the vastus lateralis from patients, a decrease in mitochondrial mRNA encoding mitofusin 1&2, which are important for mitochondrial fusion, was found in ALS patients when compared to health controls [100]. In studies utilizing muscle biopsies from ALS patients, preferential wasting is found in the gastrocnemius (GAS) muscle in comparison to the tibilialis anterior (TA), including a reduction of function in terms of plantar flexion (GAS) versus dorsiflexion (TA) [109]. However, conflicting studies emphasize the loss of the TA muscle over the gastrocnemius, proposing that the glycolytic nature of the TA is allowing the muscle to be more susceptible to atrophy over the oxidative GAS muscle [52,110]. Although conflicting data has been found regarding muscles susceptible to degeneration, the “split-leg” hypothesis is still present, although more studies must be completed in order to determine muscle susceptibility. A study looking at SOD1 mutant carriers along with cases of sporadic ALS showed a reduction in motor unit number estimate (MUNE) months before the onset of weakness, proposing a potential biomarker for earlier diagnosis [111]. Muscle biopsies from ALS patients were histochemically stained for COX; in ALS patients there was a higher number of COX-negative fibers, adding to the author’s conclusion that mitochondrial dysfunction is present in ALS patients [99].

Animal models of ALS also mimic what is seen in patient skeletal muscle tissue, similarly to what was previously reported within the CNS. In SOD1 G93A mice, with increased expression of mRNA’s allowing for the reduction of mitochondrial proteins COXIV, and peroxisome proliferator-activated receptor γ coactivator-1α (PGC-1α) emphasizing mitochondrial defects in skeletal muscle tissue [100]. Using confocal microscopy and markers for mitochondria, defects in mitochondrial dynamics were found in G93A mutant mouse in skeletal muscle samples before disease phenotype onset occurred [101,102]. Not only are morphological changes present in mitochondria in skeletal muscles, increased reactive oxygen species (ROS) have also been shown to be present in skeletal muscle tissues from SOD1 G93A mice [112]. In order to determine changes in specific muscle fiber types, a study using G93A mice determined a preferential switch from glycolytic to lipid metabolism [2]. Similarly, utilizing a different mutation, SOD1 G86R mice showed decreased glycolytic pathway activation and increased fatty acid metabolism at pre-symptomatic stages of the disease [113]. These mice also showed increased levels of pyruvate dehydrogenase kinase 4 (PDK4), allowing for the inhibition of the enzymatic activity of pyruvate dehydrogenase. These studies show the importance of documenting the stages of ALS, as time course studies emphasize changes of metabolism at varying points of the disease. Therapeutic approaches have not only included pharmaceutical intervention [52] but also utilizing varying forms of exercise, such as swimming, to ameliorate the muscular defects found at early stages of the disease [114] (Figure 1 and Table 1).

## 5. Metabolic Malfunction in Other Motor Neuron Diseases

Metabolic malfunction has been directly linked with SMA, PLS, Kennedy’s disease and HSP. Although less has been found about PLS in comparison to other NMDs, links to solely upper motor neuron loss have been demonstrated. Currently, diagnostic tests for PLS are being extensively studied in order to differentiate the disease from ALS, but preferential atrophy is found in the precentral gyrus of PLS patients, which is absent in ALS cases [115]. Along with atrophy in the same region, PLS patients have also shown hypometabolism via fluordeoxyglucose studies; however, this diagnostic marker has yet to be solidified as a standard in the field [8,103].

Although SMA has been linked to a particular gene, developing therapeutics to hinder degeneration in susceptible muscles is at the forefront of research. Specifically, studies are focusing on metabolic defects found in muscles in order to prolong their functionality. Utilizing a SMA mouse model, motor neurons showed a decrease in mitochondrial respiration, most likely from a change in mitochondrial genes found in the same model [104]. Interestingly, current studies are also focusing on the rare adult SMA, speculating that aging resulting in mitochondrial deterioration could be one of the causative factors resulting in adult-onset SMA [116]. Therefore, focusing therapeutic strategies towards malfunctioning mitochondria in aging could present one of the forms of SMA and potentially be used at earlier stages of the disease if expanded further.

Metabolic malfunction in Kennedy’s disease has been a relatively new area of study. In 2015, one group identified oxidative stress in a patient positive for the disease when compared to a female carrier and non-carrier control [117]. Knowing this disease is the result of expanded CAG repeats on the androgen receptor (AR) gene, studies have begun looking at the interactions between this expansion and mitochondrial related proteins [118]. Other studies have found that mutant AR has also been implicated in alterations of mitochondrial protein production [119]. Interestingly, although this disease is linked to a specific genetic alteration, other genes have been shown to be dysregulated. In a study using embryonic motor neurons from SBMA mice, the gene *Chmp7*, which plays a role in autophagy and endosome formation, was dysregulated before symptom onset in these mice [105]. The motor neurons derived from these animals showed a decrease in mitochondrial genes and an increase in mitochondrial malfunction, leading to dysregulated metabolism via ROS production. Although this could be a result of disease progression as a result of CAG repeat expansion, attacking other upregulated genes such as *Chmp7* could potentially ameliorate malfunctioning mitochondria and aid in hindering disease progression (Table 1).

Different HSP subtypes have been identified with mitochondrial malfunction in patient populations and in vitro studies. DDHD1 mutations have been known to increase oxidative stress by disrupting mitochondrial function in patients [120]. A study from 2016 focused on two siblings, both with the SPG28 subtype resulting from mutations in DDHD1 [106]. The results showed decreased mitochondrial DNA and changes in mitochondrial morphology via histochemical staining of both patient samples [106]. One sibling, along with cultured skin fibroblasts, showed decreased mitochondrial ATP and mitochondrial fragmentation [106]. SPG7 mutations resulting in paraplegin deficiency, a mitochondrial matrix protease, have been well-characterized and confirm reductions in mitochondrial activity [121,122]. Previous studies have evaluated SGP7 and increased paraplegin on mitochondrial morphology in patient populations but lack respiratory chain function [123]. Comparing two different HSP subtypes, SPG7 and SPAST, mitochondrial dynamics were assessed in olfactory neurosphere-derived cells from patients harboring the mutations above [107]. Only SPG7 mutants showed increased paraplegin when compared to both SPAST and healthy controls. The patient-derived SPG7 cells also demonstrated fragmented mitochondria, decreased mitochondrial membrane potential, reduced oxidative phosphorylation, reduced ATP concentration and increased oxidative stress. Mitochondrial stress shown only in SPG7 samples shows a subtype-specific disease phenotype, creating more layers in this family of diseases. SPG11 is an autosomal recessive HSP subtype that is known to bind to SPG15 and AP5, which encompasses SPG48 [124]. IPSCs from both SPG11 and SPG48 patients were differentiated into cortical projection neurons, and mitochondrial dynamics were analyzed [108]. Decreased mitochondrial length, density and ATP levels were found in both SPG11 and SPG48 when compared to healthy controls. Mitochondrial dynamics were then analyzed after induction of P110, a mitochondrial fission inhibitor, and results showed rescuing effects on all previously mentioned categories in both models. HSP is a complex family of diseases which result in numerous alterations in cellular signaling mechanisms; therefore, although there is some overlap between disease subtypes, it is not indicative of all genotypes.

## 6. Strategies to Manipulate Metabolic Dysregulation in Motor Neuron Diseases

Multiple therapeutics have been designed in order to tackle metabolic dysregulation in MNDs. A current medication on the market, riluzole, with a primary mechanism of aiding in neurotransmission, has also been shown to increase GLUT transports in vivo and increase glucose uptake in other neurodegenerative diseases as a secondary mechanism [54]. However, some therapeutics are directly targeting metabolic malfunction. Sodium Phenylbutyrate-Taurursodiol is currently in phase II clinical trials [125]. The treatment targets mitochondrial dysfunction by preventing the recruitment of pro-apoptotic protein Bax to the mitochondrial membrane, decreasing apoptosis; however, changes in mitochondrial dynamics and oxidative potential have yet to be evaluated. As previously mentioned, increasing glycolytic enzyme PFK-1 and GLUT3 resulted in a rescue of locomotive deficit found in this model, suggesting a potential therapeutic mechanism by increasing glycolytic output [92]. Similarly, a recent study has shown that increasing the mitochondrial deacetylase Sirtuin-3 results in rescuing mitochondrial respiration defects previously reported in ALS cases [94]. P110 decreased mitochondria fission and rescued mitochondrial length, density and ATP levels in iPSC models of HSP [108]. Further exploration of P110 on other subtypes of HSP should be considered moving forward. P110 has also been implemented in Parkinson’s disease models, utilizing dopaminergic neurons in order to alleviate mitochondrial fragmentation and decrease reactive oxygen species present, verifying P110 function in other diseased states [126].

Although attacking motor neurons directly has been the primary area of focus recently, targeting skeletal muscle metabolism via genetic intervention, pharmacological therapeutics or exercise modulation has gained significant traction. Currently, genetic manipulation tactics have only been utilized in SOD1 mutant mice, and although some genes increase motor function, most had no affect or even decreased survival rate [2]. Importantly, peroxisome proliferator-activated receptor-gamma coactivator-a (PGC-1a) has been the only gene manipulated with a role in aiding metabolic dysfunction and locomotive deficits, although no change in overall survival was found [127]. Currently, few pharmacological interventions have been reviewed as possible approaches to tackle metabolic changes in skeletal muscles of ALS. Dichloroacetate (DCA) is a PDK4 inhibitor resulting in increased glycolytic output and decreased fatty acid utilization, compensating for previous reports of decrease glycolytic production [52]. Another treatment, Trimetazidine (TMZ), inhibits the last step of fatty acid oxidation, thereby increasing glycolytic production [128]. Although these therapies have been tested in animal models, their use in patients for correcting metabolic dysfunction in motor neuron diseases has yet to be explored. Exercise intervention has been a heavily debated topic in the context of motor neuron diseases. Although consistent exercise has shown to aid in skeletal muscle metabolism, in the context of ALS, varying physical interventions have shown to have both positive and negative effects [2], opening the door for further investigations.

## 7. Conclusions

Motor neuron diseases show varying phenotypes at varying stages, whether genetic or sporadic in nature. Importantly, all show similar trends, with metabolic dysfunction at constant states of disease progression. This review summaries the uses of patient data and in vivo and in vitro techniques in order to better understand the metabolic changes occurring at each stage of MNDs. Currently, the field of MND therapeutics is in search of biomarkers in order to readily diagnose the disease properly, and effectively diagnose and treat before symptoms progress to late/end-stage. Targeting muscles affected by these diseases poses an opportunity to identify the disease before it has progressed to motor neurons. Combining what is known about metabolic deficits in NMDs at early, middle, and late stages, and understanding the communication between motor neurons and skeletal muscles via the NMJ, we can continue progressing the field of therapeutic intervention to target these deadly diseases.

## Figures and Tables

**Table 1:** Summary of model specific changes in metabolism found in NMDs.

Disease	Model	Findings
ALS	Neuronal—Patients	Decreased glucose uptake in motor cortex [76,77] Disrupted mitochondria in spinal cord samples [53,78,79,80,81] Increased mutated mtDNA and decreased mtDNA in cortex [82]
	Neuronal—in vivo	Defects in glucose uptake and ATP production from defective mitochondria in SOD1 models [86,87] Increased mitochondrial malfunction and altered morphology [90] Defects in mitochondrial respiration and increased oxidative stress in TDP43 mouse model [91] TDP43 *Drosophila* model increased PFK as compensatory [92]
	Neuronal—in vitro	iPSCs show increased glycolysis and decreased mitochondrial respiration [94,95] Decreased PPP intermediates resulting in oxidative stress [98]
	Skeletal—Patients	Mitochondrial dysfunction via COX-negative fibers in ALS patient muscle biopsies [99]
	Skeletal—in vivo	Reduction of mitochondrial proteins [100] Defects in mitochondrial dynamics before disease onset [101,102] Decreased glycolytic pathway activation [2]
PLS	Neuronal—Patients	Hypometabolism in the precentral gyrus [8,103]
SMA	Neuronal—in vivo	SMA mouse model shows decreased mitochondrial respiration [104]
Kennedy’s Disease	Neuronal—in vitro	Decreased mitochondrial genes and increased ROS [105]
HSP	Skeletal—Patients	SPG28 subtype decreased mitochondrial DNA and altered mitochondrial morphology [106]
	Neuronal—in vitro	SPG7 subtype olfactory neurosphere-derived cells demonstrated fragmented mitochondria, decreased mitochondrial membrane potential, reduced oxidative phosphorylation, reduced ATP concentration and increased oxidative stress [107] IPSCs derived from SPG11 and SPG48 subtypes show decreased mitochondrial length, density and ATP levels [108]

## Data Availability

No new data were created or analyzed in this study. Data sharing is not applicable to this article.
